# Radiotherapy of Breast Cancer in Laterally Tilted Prone vs. Supine Position: What about the Internal Mammary Chain?

**DOI:** 10.3390/jpm12040653

**Published:** 2022-04-18

**Authors:** Nils Temme, Robert Michael Hermann, Tanja Hinsche, Jan-Niklas Becker, Mathias Sonnhoff, Alexander Kaltenborn, Ulrich Martin Carl, Hans Christiansen, Lilli Geworski, Mirko Nitsche

**Affiliations:** 1 Radiologie Munchen, 80333 Muenchen, Germany; n.temme@strahlentherapie-muenchen.eu; 2 Center for Radiotherapy and Radiooncology Bremen and Westerstede, 26655 Westerstede, Germany; hinsche@strahlentherapie-nord.com (T.H.); sonnhoff@strahlentherapie-westerstede.com (M.S.); carl@strahlentherapie-nord.com (U.M.C.); nitsche@strahlentherapie-nord.com (M.N.); 3 Department of Radiotherapy and Special Oncology, Hannover Medical School, 30625 Hannover, Germany; strahlentherapie@mh-hannover.de (J.-N.B.); christiansen.hans@mh-hannover.de (H.C.); 4 Department of Trauma and Orthopaedic Surgery, Section for Plastic Reconstructive and Hand Surgery, Federal Armed Forces Hospital Westerstede, 26655 Westerstede, Germany; alexander.kaltenborn@gmx.de; 5 Department for Radiation Protection, Hannover Medical School, 30625 Hannover, Germany; geworski.lilli@mh-hannover.de; 6 Radiotherapy, Karl-Lennert-Krebscentrum, Universität Kiel, 24105 Kiel, Germany

**Keywords:** radiotherapy, breast cancer, prone position, supine position, internal mammary chain, parasternal region, IMRT, plan quality

## Abstract

Background: In the multimodal breast-conserving curative therapy of some high-risk breast cancer patients, extended external beam radiotherapy (EBRT) not only to the breast but also to the supraclavicular fossa and the internal mammary chain (parasternal region (PSR)) is indicated. We report a dosimetric study on the EBRT of the breast (“B”) and the breast including PSR (“B + PSR”), comparing the supine and the laterally tilted prone patient positions in free breathing. Methods: The planning CT scans of 20 left- and 20 right-sided patients were analyzed. EBRT plans were calculated with 3D conformal EBRT (3D) and with intensity-modulated EBRT (IMRT) for “B” and “B + PSR” in the prone and supine positions. The mean and threshold doses were computed. The quality of EBRT plans was compared with an overall plan assessment factor (OPAF), comprising three subfactors, homogeneity, conformity, and radiogenic exposure of OAR. Results: In the EBRT of “B”, prone positioning significantly reduced the exposure of the OARs “heart” and “ipsilateral lung” and “lymphatic regions”. The OPAF was significantly better in the prone position, regardless of the planning technique or the treated breast side. In the EBRT of “B + PSR”, supine positioning significantly reduced the OAR “heart” exposure but increased the dose to the OARs “ipsilateral lung” and “lymphatic regions”. There were no significant differences for the OPAF, independent of the irradiated breast side. Only the IMRT planning technique increased the chance of a comparatively good EBRT plan. Conclusion: Free breathing prone positioning significantly improves plan quality in the EBRT of the breast but not in the EBRT of the breast + PSR.

## 1. Introduction

A mainstay of modern breast-conserving surgery (BCS) for breast cancer is adjuvant external beam radiotherapy (EBRT). EBRT following BCS reduced recurrence rates between one-third to one-half compared to BCS alone in large studies with longtime follow-up (EBCTCG) [1]. In nodal positive breast cancer, overall survival is even increased by 8% after 15 years. Actually, after a complete histopathological response to modern neoadjuvant systemic therapies, adjuvant EBRT is still indispensable. 

Traditionally, the whole breast is treated. However, in patients with low-risk tumors, partial breast irradiation might be as effective [2]. In contrast, in high-risk patients, extended EBRT not only to the breast but also to the supraclavicular fossa and to the internal mammary chain (in the following called the parasternal region (PSR)) increased long-term overall survival by 1–2% and disease-specific survival by 8% after ten years in three randomized trials [3].

Conventionally, adjuvant EBRT has been planned as a 2D, nowadays as a 3D, tangential field arrangement with dose homogenization (wedges, additionally individually planned fields, or intensity-modulated radiotherapy planning (IMRT)). The patient is placed in the supine position. This technique results mostly in good dose distributions. However, when EBRT is extended to the PSR in high-risk patients with a “wide tangent”, the radiation exposure of the lung and heart is substantially increased, especially in left-sided cancers. Thus, the benefits of EBRT could be outweighed by the risks of causing damage to these organs.

To overcome this obstacle, new techniques have been developed and implemented into the clinical routine. One possibility is to apply the so-called deep inspiration breath-hold technique: by the expansion of the thorax, the distance between the treatment volume and the heart increases, thus reducing the mean heart dose [4]. However, a substantial lung dose is still unavoidable.

Another option is to place the patient for EBRT not in the standard supine position but in the prone position. Some devices allow for a rotation of the patient on the affected side by several degrees (“laterally tilted prone position”). Such a technique improves the conformality of EBRT in most patients, thus reducing dose exposure not only to the lung but also to the heart [5]. However, there are limited data on the influence of patient positioning during the EBRT of the breast together with the PSR [6,7].

In the presented planning study, we compare the effects of different patient positioning (prone vs. supine) and different radiation planning techniques (3D conformal vs. IMRT) on EBRT conformity, the radiogenic exposure of the organs at risk (lung and heart), and plan quality for both EBRT of the breast (in the following called the PTV “B”) and EBRT of the breast including the PSR (the PTV “B + PSR”).

## 2. Materials and Methods

The planning study was approved by the local ethic committee (Medizinische Hochschule Hannover, Nr. 9758_BO_K_2021). All patients gave their permission to use their anonymized CT slices for research purposes.

After BCS, planning CT scans of 20 consecutive left-sided and 20 right-sided breast cancer patients with node-negative tumors who presented to our institutions for adjuvant EBRT were analyzed. All patients received treatment-planning CT scans in supine and prone positions in free breathing (no specific control of breath cycle). In the supine position, patients were placed on the “Universalboard 2000” (Unger, Erfurt, Germany), whereas in the prone position, patients were laid down on the Sagittilt (Orfit, Wijnegem, Belgium) and were rotated 7° in the direction of the affected side (the so-called “laterally tilted prone position”). Five mm CT-scans for both prone and supine positions were obtained by a CT scanner (Sensation Open, Siemens, Erlangen, Germany).

All CT scans were contoured and planned for the study independently from the clinically applied EBRT plans. In Pinnacle3 (Philips, Eindhoven, Netherlands), additional to the organs at risk (OAR), heart and lung, two clinical target volumes (CTV) were defined, based on the ESTRO-Guidelines in the supine position [8]: (a) the whole breast (CTV “B”) and (b) the internal mammary chain (CTV “B + PSR”). As no internationally accepted contouring guidelines were available for the prone position, contouring was performed accordingly. The CTVs were expanded by 5 mm in all directions (with a 5 mm distance to the body contour) to create the corresponding planning target volumes (PTV “B” and PTV “B + PSR”). Furthermore, we defined the lymph nodes axillary levels I–III and Rotter lymph nodes as regions of interest.

We used two different techniques for treatment planning: (a) 3D conformal radiotherapy (3D) and (b) intensity-modulated radiotherapy (IMRT). A collapsed-cone algorithm was applied. The treatment plans were calculated for a linear accelerator (Siemens, Erlangen, Germany) with a 160-leaf collimator and 6 MV/15 MV photon energies. In total, we calculated eight treatment plans per patient: 4 each in the supine and in the prone positions, for both a 3D and an IMRT plan for PTV “B” and PTV “B + PSR”.

**Figure 1:**
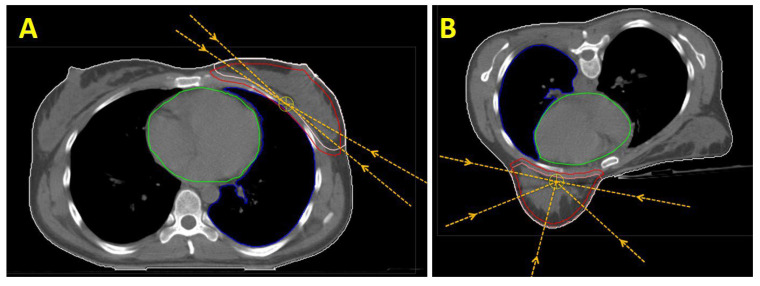
(**A**): Illustration of the main EBRT directions for the IMRT technique for treating the breast (PTV “B”) in the supine position (“tangential IMRT”). Also shown is the contour of the CTV “Breast” (light red) and PTV “B” (red), as well as the OARs “heart” (green) and ipsilateral lung (blue). (**B**): Main EBRT directions for the IMRT technique for treating the breast in a laterally tilted prone position (“multibeam IMRT”).

For 3D planning, we used a tangential technique in the prone and the supine positions, deploying 6 MV photon main beams and extra 15 MV photon beams for homogeneity. In IMRT planning of PTV “B”, we only used 6 MV: in the prone position, a multibeam IMRT approach with about 30 segments, and in the supine position, a tangential IMRT with about 30 segments (Figure 1). For covering PTV “B + PSR”, IMRT consisted of up to 45 segments.

A single dose of 1.8 Gy up to a total dose of 50.4 Gy was prescribed. We did not use a hypofractionated EBRT concept, as normofractionated EBRT is currently still the standard for treating parasternal lymph nodes in Germany. In addition, we refrained from planning the irradiation of the supraclavicular lymph nodes or of tumor bed boosts in order to be able to more clearly illustrate the effect of the coverage of the PSR concerning the exposure of the lungs and various axillary lymph node regions. 

We calculated all plans according to the ICRU guidelines. For 3D conformal planning, the target volume concepts and dose specifications defined in ICRU report 50 were used. Here, 100% of the PTV had to have received at least 95% of the dose and 0% of the volume more than 107% of the dose. For IMRT radiation planning, we applied the specifications of ICRU report 83. Again, coverage between 95 and 107% of the prescribed dose had to be analyzed, but the lower and upper 2% of the volume values were not considered. This was performed so that individual voxels were not given too much weight during optimization during radiation planning. To be more precise: 98% of the volume had to receive at least 95% of the dose (D98), and 2% were allowed to receive more than 107% (D2).

To evaluate the calculated treatment plans, we calculated the mean dose for each volume. Furthermore, we set threshold doses for each volume: (a) OAR “lung”: volume receiving at least 20 Gy (V20); (b) OAR “heart” and ROI “lymph nodes”: volume receiving at least 40 Gy (V40).

To compare the quality of EBRT plans within the two anatomical planning situations (PTV “B” or PTV “B + PSR”), we developed a plan assessment factor comprising three equally weighted subfactors:

(a) *The homogeneity of the dose distribution in the PTV*: This is calculated from the relative difference of the PTV volume, which was exposed to 48 Gy or 52 Gy (approximately 95% or 103% of the PTV dose). Therefore, the value lay between 0 and 100; the higher it was, the more homogeneous the dose distribution in the target volume was. Subsequently, we determined a mean value from all irradiation plans. If the value of an individual plan was higher than the mean value, the irradiation plan received a factor of “1” for homogeneity; otherwise, a factor of “0”.
(b) *The conformity of the dose distribution*: This describes whether the plan primarily covered the PTV and produced a steep dose fall-off in the surrounding normal tissues. It was calculated from the *absolute* volume outside the PTV + 5 mm exposed to 10 Gy and to 48 Gy (V10 and V48). These parameters were compared with the mean value of the corresponding collective. If the value for the respective plan was lower than the mean value of the collective, the surrounding normal tissue was less exposed in comparison to the entire collective; the irradiation plan received a 1; otherwise, a 0. The partial values for V10 and V48 were added together and divided by two so that the partial factor conformity resulted in between 0 and 1.
(c) *The radiogenic exposure of OAR*: Here, both the average dose exposures and the organ-specific dose thresholds (as described above) were considered. The value was composed of the mean lung dose, the lung V20, the mean cardiac dose, and the cardiac V40. If the risk structures in the respective plan were exposed to less than the average of the corresponding collective, the result for this subfactor was “1”; otherwise, “0”. To calculate the subfactor “radiogenic exposure of OAR”, the sum of the individual OAR factors was then divided by the total number of factors, resulting in a value between 0 and 1.

The individual three subfactors were added together and divided by three to determine the overall plan assessment factor (OPAF). This also resulted in a value between 0 and 1, whereby the plan quality increases with the value. Thus, “1” stands for an excellent treatment plan, while “0” indicates a plan that poorly fulfills the planning goals.

### Statistical Methods

We assessed all continuous data for normal distribution applying the Kolmogorov–Smirnov Test (Statistica 10, Stat Soft, Tulsa, OK, USA). If this test was significant (*p* < 0.05), the data were not normally distributed. Normally distributed data were presented as mean with standard deviation and were analyzed with the paired Student’s *t*-Test. 

Possible relevant factors (prone vs. supine; 3D vs. IMRT; the volume of the PTV “Breast” <600 cm^3^ vs. 600–900 cm^3^ vs. >900 cm^3^) on planning quality were identified by univariate binary logistic regression analyses applying an alpha-level of 0.20. Hence, all variables with a *p*-value < 0.20 were included in multivariate regression analyses. In multivariate analyses, a *p*-value < 0.05 was defined as statistically significant. We used the SPSS statistics software 23.0 (IBM, Somers, NY, USA) to perform these statistical analyses.

## 3. Results

Clinicodemographic characteristics are given in Supplementary Tables S1 and S2. The mean age of the patients was 53.5 years (range 29–84). Breast volume in the prone position ranged from 204 cm^3^ to 1520 cm^3^ (mean 764 cm^3^, SD 290) and in the supine position from 179 cm^3^ to 1419 cm^3^ (mean 766 cm^3^, SD 296).

### 3.1. EBRT of PTV “B” (Treating Only the Breast) 

Table 1 summarizes all results for the OARs and further ROIs. 

#### 3.1.1. Organs at Risk

- *OAR “heart” in the EBRT of the left breast*

Due to its left lateral position in the thorax, the heart only represents a risk in EBRT for left-sided tumor localization, not in the EBRT of the right breast. 

In the prone position, the heart was less exposed to radiation, irrespective of the applied planning technique, than in supine position (Supplementary Figure S1): prone 3D, 2.8 Gy (SD 1.02 Gy) vs. supine 3D, 4.76 Gy (SD 2.18 Gy), *p* < 0.001; prone IMRT, 3.36 Gy (SD 0.56 Gy) vs. supine IMRT, 4.16 Gy (SD 1.3 Gy), *p* = 0.008.

Accordingly, the cardiac volumes exposed to high doses (at least 40 Gy: V40) were significantly lower in the prone position than in the supine (*p* < 0.001 regardless of the planning technique); for details, cf. Supplementary Figure S2.

- *OAR “ipsilateral lung”*

Similar to the heart, the prone position led to an impressive reduction in the radiation dose in the adjacent lung. In the prone position with 3D, the mean lung dose was 1.74 Gy (SD 1.05 Gy) and 2.81 Gy (SD 0.72 Gy) with IMRT. The mean dose increased to 8.06 Gy (SD 1.55 Gy, 3D) and 8.0 Gy (SD 1.3 Gy, IMRT) in the supine position. Detailed results for left-sided and right-sided tumors are given in Table 1 and Supplementary Figure S3.

Comparably, the endpoint V20 reflects the significant relief of the ipsilateral lung from radiation by prone patient positioning (Table 1).

#### 3.1.2. Lymph Node Areas

In order to test the conformity of the EBRT, i.e., the ability to cover the PTV alone but not the adjacent anatomical regions, the various lymph node areas directly adjacent to the breast were contoured and analyzed as “regions of interest” (ROIs).

- *Axillary levels*

The mean dose in the axillary lymphatic regions was dramatically reduced in the prone position compared to the supine position (*p* < 0.001 for each planning technique). For example, level I prone 3D resulted in a mean dose of 13.23 Gy (SD 8.65 Gy) and IMRT in a mean dose of 16.6 Gy (SD 6.87 Gy). However, supine 3D had a mean dose as high as 33.01 Gy (SD 10.25 Gy) and IMRT as high as 33.57 Gy (SD 9.46 Gy) (Supplementary Figure S4). The analysis of the high-dose areas (V40) within this lymph node region underlined the above results (Supplementary Figure S5).

Similar results were seen in level II, although here, of course, only a small “accidental” dose was applied in general (still with a significant reduction in dose exposure with *p* < 0.001) (Supplementary Figure S6): in prone position 3D, the mean dose was 1.75 Gy (SD 3.16 Gy), while IMRT led to a slightly higher exposition with 4.55 Gy (SD 3.26 Gy). In comparison, the supine position resulted with 3D planning in a mean dose of 13.01 Gy (SD 12.03 Gy) and with IMRT in a mean dose of 14.55 Gy (SD 12.08 Gy).

Predictably, the dose exposures in level III were even lower, but again the same ratios were seen as in the other axillary levels (*p* < 0.001) (Supplementary Figure S7): the mean dose 3D planning in prone position resulted in 1.45 Gy (SD 1.65 Gy) and IMRT resulted in in 4.12 Gy (SD 3.13 Gy). In the supine position, 3D planning led to 10.17 Gy (SD 10.97 Gy) and IMRT led to 12.08 Gy (SD 11.57 Gy).

- *Rotter lymph nodes (interpectoral nodes)*

Comparable to the results in the axillary lymph nodes, the dose in the Rotter lymph nodes was also significantly reduced by prone positioning. The mean dose in the prone position was 15.75 Gy (SD 10.78 Gy) in 3D conformal planning and 19.95 Gy (SD 8.81 Gy) with IMRT. However, in the supine position, the mean doses were much higher: 35.57 Gy (SD 11.19 Gy, 3D conformal) and 35.96 Gy (SD 11.56 Gy, IMRT) (Supplementary Figure S8). The same result was obtained in the analysis of high-dose volumes (V40) (Data on interpectoral nodes, V40 (%) are given in Table 1).

- *Internal mammary chain (parasternal region, PSR)*

Also in this region, significantly higher radiogenic exposures were shown for the supine position (Supplementary Figure S9). The mean dose in the parasternal lymph nodes for 3D planning in the prone position was 9.67 Gy (SD 7.11 Gy) and the mean dose was 11.8 Gy (SD 6.18 Gy) for IMRT. In the supine position, by contrast, there were significantly higher exposures of 16.49 Gy (SD 10.37 Gy) for 3D and 17.32 Gy (SD 9.37 Gy) for IMRT.

#### 3.1.3. Plan Assessment Factors and Plan Rating

The subfactor “homogeneity” could reach a value between 0 and 1 (whereby plan quality increased with the value). It achieved a mean value of 0.03 (SD 0.16) for 3D and 0.3 (SD 0.46) for IMRT in the prone position (Table 2). In the supine position, however, significantly higher values of 0.78 (SD 0.42, 3D) and 0.98 (SD 0.16, IMRT) were reached. This subfactor thus benefited significantly from the supine position (*p* < 0.001 in each case).

The subfactor “conformity” achieved a mean value of 0.85 (SD 0.26) in the prone position for 3D and 0.78 (SD 0.25) for IMRT. In the supine position, however, values of 0.4 (SD 0.46, 3D conformal) and 0.33 (SD 0.38, IMRT) were obtained. Thus, the conformity was significantly better in the prone position for both irradiation techniques (*p* < 0.001 in each case).

The radiogenic exposure of OARs resulted in mean subfactors of 0.96 (SD 0.13, 3D) and 0.98 (SD 0.07, IMRT) in the prone position compared to 0.08 (SD 0.17, 3D) and 0.06 (SD 0.16, IMRT) in the supine position. For this subfactor, as with conformity, the prone position also showed significantly better results, regardless of the planning technique (*p* < 0.001 in each case).

No differences were found between left- and right-sided irradiation plans (data not shown).

All three subfactors were subsumed in the overall plan assessment factor (OPAF). For the whole group of left- and right-sided patients in the prone position, the OPAF reached 0.61 (SD 0.1) with 3D planning and 0.69 (SD 0.18) with IMRT. The OPAF was significantly lower in the supine position, with values of 0.42 (SD 0.26, *p* < 0.001, 3D) and 0.45 (SD 0.15, *p* < 0.001, IMRT), respectively. Thus, the overall plan quality benefited from the prone position.

The binary logistic regression showed a significant favorable influence on OPAF for planning on PTV “B” by prone positioning and a PTV “B” below 600 cm^3^. Prone positioning increased the chance of a “good plan” sixfold (RR (relative risk) 6.53, CI 95%, 3.23–13.2, *p* < 0.001)). A small PTV “breast volume” (V< 600 cm^3^) doubled this chance (RR 2.29 (CI 95%, 1.01–5.18, *p* < 0.046)). On the other hand, the planning technique had no significant influence on the OPAF. Results of the multivariable analyses are given in Supplementary Table S3.

### 3.2. EBRT of PTV “B + PSR” (Treating Breast and Parasternal Lymphatics) 

In these analyses, the results were calculated for the collective of all patients, if not described differently. Table 3 summarizes all results for OARs and further ROIs.

#### 3.2.1. Organs at Risk

- *OAR “heart”*

When planning for PTV “B + PSR”, the heart represented a clinically relevant OAR in the entire collective due to the anatomical location of the parasternal lymph nodes near the heart.

Accordingly, the mean cardiac exposures in the collective of the left breast irradiated in the prone position were 7.24 Gy (SD 2.23 Gy) for 3D and 7.35 Gy (SD 1.78 Gy) for the IMRT technique. In the supine position, the mean values were 7.3 Gy (SD 2.9 Gy) for 3D and 6 Gy (SD 2.02 Gy) for IMRT. Here, IMRT computed significantly less dose exposure in the supine position than in the prone position (Supplementary Figure S10).

However, when the heart volume exposed to a high dose (V40) was considered, there was no longer an advantage for the supine position (Supplementary Figure S11). The V40 values in the prone position were 7.19% (SD 3.39%, 3D) and 2.53% (SD 1.53%, IMRT), in comparison to 7.87% (SD 4.54%, 3D) and 3.77% (SD 2.41%, IMRT) in the supine position, i.e., the lowest V40 was achieved by IMRT planning in the prone position.

For the EBRT of the right-sided breast, including PSR, there were significant advantages for the supine position—independent of the planning technique (Supplementary Figure S12). For the prone position, mean values of 1.8 Gy (SD 0.82 Gy) were calculated for 3D and 3.54 Gy (SD 1.19 Gy) for IMRT. On the other hand, in the supine position, the mean cardiac exposures were 1.07 Gy (SD 0.42 Gy, 3D) and 1.61 Gy (SD 0.94 Gy, IMRT). These results were also obtained for the right breast by analyzing the high-dose volumes (V40), with a significantly lower mean value in 3D planning for the supine position than for the prone position (*p* = 0.008, Table 3).

Consequently, the heart was significantly less exposed in the supine position with the IMRT planning technique in both collectives (right- and left-sided EBRT) than in the prone position (left: *p* = 0.015, right: *p* < 0.001). However, there was no significant advantage for 3D planning of the left breast due to positioning (*p* = 0.47). For the EBRT of the right breast, on the other hand, the supine position led to significant protection in 3D planning (*p* < 0.001).

- *OAR “ipsilateral lung”*

Like EBRT for the PTV “B“, the lung dose was much higher in the supine position.

For the left breast, the mean lung doses in the prone position were 7.42 Gy (SD 2.53 Gy) in 3D and 7.52 Gy (SD 1.72 Gy) in IMRT. However, in the supine position, the mean doses were as high as 13.12 Gy (SD 2.6 Gy, 3D) and 12.65 Gy (SD 0.99 Gy, IMRT). The V20 values for the left lung were 14.44% (SD 5.38%, 3D) and 15.21% (SD 5.62%, IMRT) in the prone position, in comparison to 30.44% (SD 6.22%, 3D) and 28.51% (SD 5.02%, IMRT) in the supine position.

Similar results were obtained for the EBRT “B + PSR” of the right side (Table 3). Data for the entire collective are given in Supplementary Figure S13.

#### 3.2.2. Lymph Node Areas

- *Axillary levels*

In the prone position, the axillary level I mean doses were 31.8 Gy (SD 8.58 Gy) in 3D and 26.79 Gy (SD 6.76 Gy) in IMRT. However, in the supine position, the mean doses were significantly higher, with 35.95 Gy (SD 8.19 Gy, *p* = 0.015) in 3D and 33.57 Gy (SD 9.46 Gy, *p* < 0.001) in IMRT (Supplementary Figure S14).

The analysis of the high-dose area (V40) showed similar results: In the prone position, the V40 values were 51.11% (SD 20.47%, 3D conformal) and 24.4% (SD 16.94%, IMRT). However, in the supine position, the V40 values increased significantly to 62.05% (SD 18.99, 3D, *p* = 0.007) and 61.03% (SD 17.97, IMRT, *p* < 0.001) (Supplementary Figure S15).

Similar results were obtained for the level II lymphatic region: In the prone position, the radiogenic exposures were significantly lower, with 8.21 Gy (SD 7.32, 3D) and 10.53 Gy (SD 6.86, IMRT) in comparison with the supine position: 13.03 Gy (SD 10.58, 3D, *p* = 0.01) and 16.14 Gy (11.09, IMRT, *p* = 0.004) (Supplementary Figure S16).

Even in level III, the radiogenic exposure significantly favoured the prone position: In this lymphatic region, the mean doses increased from 6.74 Gy (6.67, 3D) and 9 Gy (SD 5.98, IMRT) in the prone position to 10.96 Gy (SD 9.05, 3D, *p* = 0.01) and 14.32 Gy (SD 10.76, IMRT, *p* = 0.004 in the supine position (Supplementary Figure S17).

- *Rotter lymph nodes (interpectoral nodes)*

The mean doses of the Rotter lymph nodes in the prone position were 26.2 Gy (SD 10.43) in 3D and 24.6 Gy (SD 7.82) in the IMRT technique. In the supine position, the mean doses increased significantly to 35.53 Gy (SD 11.14, 3D, *p* < 0.001) and 36.76 Gy (SD 10.84, IMRT, *p* < 0.001) (Supplementary Figure S18).

Similar ratios were calculated for the high-dose-area values (V40). They increased from 40.45% (SD 23.99, 3D) and 23.31% (SD 19.11, IMRT) in the prone position to 66.77% (SD 23.82, 3D, *p* < 0.001) and 69.33% (SD 23.1, IMRT, *p* < 0.001) in the supine position, respectively.

#### 3.2.3. Plan Assessment Factors and Plan Rating

The subfactor “homogeneity” achieved a mean value of 0.35 (SD 0.26) for 3D and 0.44 (SD 0.32) for IMRT in the prone position (Table 4). In the supine position, however, significantly higher values of 0.56 (SD 0.23, 3D, *p* < 0.001) and 0.61 (SD 0.33, IMRT, *p* = 0.009) were reached. This subfactor thus benefited significantly from the supine position.

The subfactor “conformity” achieved a mean value of 0.41 (SD 0.36) in the prone position for 3D and 0.59 (SD 0.25) for IMRT. In the supine position, values of 0.56 (SD 0.41, 3D) and 0.69 (SD 0.39, IMRT) were obtained. Thus, the conformity was significantly increased by the supine position in 3D planning (*p* = 0.042), but it was comparable between both positionings for IMRT (*p* = 0.12).

The radiogenic exposure of OAR resulted in a mean subfactor of 0.64 (SD 0.31, 3D) and 0.73 (SD 0.19, IMRT) in the prone position compared to 0.42 (SD 0.3, 3D) and 0.43 (SD 0.23, IMRT) in the supine position. The prone positioning showed significantly better results for this subfactor, regardless of the planning technique (3D, *p* = 0.001; IMRT, *p* < 0.001).

All three subfactors were subsumed in the overall plan assessment factor (OPAF, a value between 0 and 1, with plan quality increasing with value). For the whole collective of left- and right-sided patients in the prone position, the OPAF reached 0.47 (SD 0.18, 3D) and 0.59 (SD 0.17, IMRT) without significant differences in comparison with the supine position: 0.51 (SD 0.2, 3D, *p* = 0.131) and 0.58 (SD 0.22, IMRT, *p* = 0.4). No significant differences were found between the left- and right-sided irradiation plans in terms of OPAF (Table 4).

The binary logistic regression showed a significant favorable influence on OPAF for IMRT planning compared to 3D planning. IMRT doubled the chance for a good planning result (RR 2566 (CI 95%, 1.31–5.001, *p* = 0.006)). However, positioning (prone vs. supine) and the volume of PTV had no significant influence on OPAF. The results of the multivariable analyses are given in Supplementary Table S4.

### 3.3. Summary of Results

**In the EBRT of PTV “B”,** positioning in the prone position led to a significantly reduced exposure of the OARs “heart” and “ipsilateral lung”. Furthermore, the conformity of the EBRT plans in the prone position was significantly higher than in the supine position (Table 5).

The OPAF was significantly better in the prone position than in the supine position, regardless of the planning technique used. Although the supine position performed better in the subfactor “homogeneity”, the prone position achieved significantly higher values for the subfactors “conformity” and “radiogenic exposure of OAR”. These observations were independent of the irradiated breast side.

**In the EBRT of PTV “B + PSR”,** supine positioning led to significantly reduced exposure of the OAR “heart”. Only the IMRT technique in the prone position resulted in a lower mean cardiac V40 value for left-sided irradiation than in the supine position. 

In contrast, the ipsilateral lung was significantly less exposed in the prone position, despite the inclusion of the parasternal lymph nodes. Likewise, the lymphatic regions received a significantly smaller dose in the prone position, analogous to the EBRT PTV “B” results.

Considering the plan assessment factors, no significant difference between the positions was found. Even though the prone position performed significantly better for the subfactor “radiogenic exposure of OAR”, the supine position provided significantly higher values for homogeneity and conformity. Taken together, there were no significant differences for the OPAF, independent of the irradiated breast side.

The binary or logistic regression also reflects the fact that no clear statement can be made regarding the positioning for the planning on the PTV “B + PSR”. Thus, only the IMRT planning technique increases the chance of a comparatively good EBRT plan and not the positioning itself.

## 4. Discussion

### 4.1. OAR “Heart”

The heart is a critical OAR in breast EBRT. McGale et al. published data on more than 34.000 women irradiated for breast cancer between 1976 and 2006 with long-term follow-up [9]. The mean heart dose was 6.3 Gy for left-sided and 2.7 Gy for right-sided cancers. The incidence ratios for left-sided EBRT were significantly increased up to 1.22 for acute myocardial infarction (95% CI, 1.06–1.42), 1.25 for angina (1.05–1.49), 1.61 for pericarditis (1.06–2.43), and 1.54 for valvular heart disease (1.11–2.13). Further analysis by Darby et al. described a linear dependence of the risk on the mean heart dose, with the risk of a major coronary event increasing by 7.4% per Gray mean heart dose [10].

Our work shows that the EBRT of the PTV “B” in a laterally tilted prone position significantly reduces the mean heart dose, regardless of the planning technique used. In addition, we found a lower V40 value (as a surrogate for the high dose) by the prone position and associated higher conformity.

The literature partially supports these findings. For example, Veldemann et al. also described a significantly lower mean cardiac dose by a laterally tilted prone position [11]. In addition, Kirby et al. found a reduction in the mean cardiac dose in the prone position, at least for women with large breast volume [12]. Other studies, however, could not show any cardiac protection [13,14,15]. Different positioning systems can explain these differing findings: the studies with negative results lacked the possibility of rotation around the longitudinal axis, in comparison to our study and that of Veldemann et al. This results in disadvantages in planning because the contralateral breast as a limiting factor prevents the flat, heart-sparing directions of the EBRT beams. 

**Figure 2:**
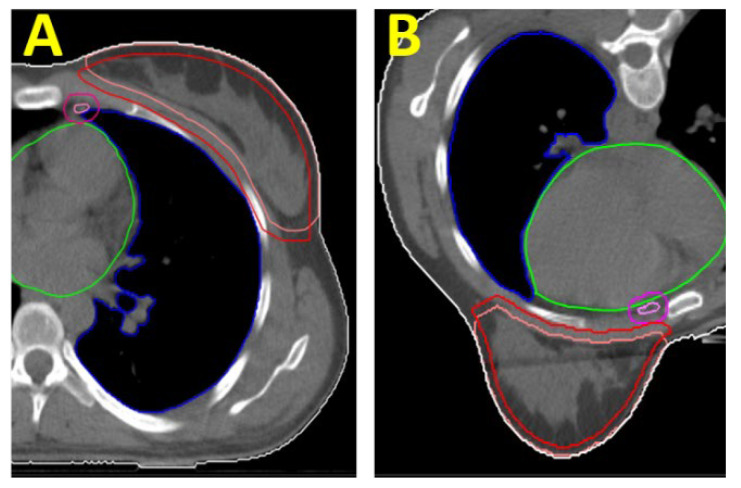
CT sections of a patient with left-sided breast cancer: (**A**) in the supine and (**B**) in the prone positions. Contours marked are the CTV “B” (light red), the PTV “B” (red), the CTV “PSR” (pink), and the PTV “PSR” (purple). In addition, the OAR “heart” (green) and OAR “left lung” (blue) are depicted.

In contrast, for the EBRT of the PTV “B-PSR”, there was no advantage of the prone position over the supine position in our study. In the prone position, gravity pulls the heart ventrally towards the sternum and thus towards the caudal parasternal lymph nodes. This inevitably results in closer proximity of the heart to the PTV and the high-dose area (Figure 2). Consequently, the cardiac dose is higher in the prone position. Even if the difference is not so pronounced for patients affected on the left side due to the left-lateral position of the heart (mean heart dose in the prone position was 7.35 Gy and in the supine position was 6.0 Gy), the effect is considerable for patients irradiated on the right side. Taken together, the supine position results in significantly lower cardiac exposure in the supine position.

Our calculated mean cardiac dose for the EBRT of the “left breast + PSR” in the supine position between 6 Gy (IMRT) and 7.3 Gy (3D) is supported by the literature with a mean dose of 8 Gy [16]. However, cardiac exposure is substantially decreased by deep inspiration breath-hold techniques, i.e., from 6.2 Gy to 3.1 Gy [17]. Only one planning study has investigated the EBRT of the PTV “B + PSR” in free-breathing prone position, to the best of our knowledge. Speleers et al. positioned ten patients in a “prone crawl” device developed for proton beam RT [6]. With IMRT/VMAT photon planning, the average mean heart dose in the supine position was 5.6 Gy (40.05 Gy total dose, about 7 Gy for 50 Gy total dose), in the prone position 4.3 Gy (5.38 Gy calculated for 50 Gy). In our study, the mean heart dose was slightly higher, with 7.35 Gy, probably due to step-and-shoot IMRT planning in our study vs. VMAT. Speelers et al. demonstrated the best planning results for pencil beam scanning intensity-modulated *proton* therapy (IMPT) with 1 GyRBE irrespective of positioning [6]. The same group showed a further dose reduction to the heart by DIBH in the prone position [7]. However, these techniques are highly complex and are only available in a few places worldwide. Unfortunately, this means that they do not play a relevant role in the clinical care of most breast cancer patients.

### 4.2. OAR “Lung”

Besides the heart, the lungs are the most important organ at risk during and after EBRT for breast carcinoma. Firstly, a higher radiation dose is associated with a risk of developing radiation pneumonitis. At a V20 < 30%, the risk is about 6%, but at a mean of 35%, it is increased to about 30% [18]. Secondly, EBRT increases the risk of second cancer in the lung: In a large analysis of about 50,000 patients and decades of follow-up, the hazard ratio was 1.27 (1.04–1.55) [19]. A meta-analysis showed a significant increase in the risk of lung cancer after ten years, especially in active smokers. After 20 years, it increased from approximately 3.8% to 4.5% due to 5 Gy mean lung dose; after 30 years, from 9.4% to 13.8% [20].

Our data show that positioning in a laterally tilted prone position significantly reduces lung exposure compared to the supine position in the EBRT of the PTV “B”, regardless of the planning technique used and the side affected. These findings are confirmed by the literature [11,12,15].

Furthermore, our data show that with the EBRT of the PTV “B + PSR”, the radiogenic exposure of the ipsilateral lung is inevitably increased since the PTV “PSR” includes part of the lung. However, even for this situation of EBRT planning, the mean dose of the lung in the prone position is significantly lower than in the supine position, regardless of the planning technique used.

The literature supports our findings concerning the values for the EBRT of the PTV “B + PSR” in the supine position [21,22,23]. However, Speleers et al. calculated for IMRT/VMAT planning an average mean lung dose of 5.91 Gy (7.4 Gy for a total dose of 50 Gy) in the supine and 2.9 Gy (3.6 Gy) in the “prone crawl” positions [6]. Our results are significantly higher, with 12.6 Gy (supine) and 7.5 Gy (prone). The most likely explanation for this discrepancy seems to be the different IMRT planning techniques used (step-and-shoot in our study vs. VMAT).

### 4.3. Lymphatic Regions

The EBRT of the lymph node areas draining the female breast is a much-discussed topic. In the standard technique with “tangents” for whole-breast irradiation (WBI), the lower axillary levels are not included in the target volume definition, but they are accidentally covered by a tumoricidal radiation dose [24]. 

The resulting oncological effects are discussed controversially in the literature. For example, the results of the ACOSOG Z011 study, which omitted a secondary axillary dissection in patients with a maximum of two affected sentinel lymph nodes (without gross extranodal disease), were attributed to the accidental irradiation of the lower axillary lymph node levels during WBI [25,26]. The AMAROS study directly compared a secondary axillary dissection with the EBRT of the axillary and supraclavicular lymph node areas in early carcinomas with clinically negative lymph nodes but histologically affected sentinel lymph nodes [27]. With similar oncological results, EBRT was associated with less toxicity, especially concerning lymphoedema of the arm.

On the other hand, axillary SNLE is such an accurate staging examination (at least in patients without prior systemic therapy) that any other therapy of the axilla can be omitted oncologically in early node-negative breast carcinomas [28]. Therefore, for radiation protection and toxicity reduction, this region must not be included in EBRT if technically possible. In these collectives, smaller patient series did not show increased local or regional recurrence rates [29,30,31].

We show that in the EBRT of the PTV “B”, the adjacent lymph node areas (axillary, interpectoral, and parasternal) are exposed to significantly smaller doses in the prone position than in the supine position, regardless of the planning technique used. This is mainly due to the high conformity of the dose distribution in the prone position. Several other studies support these results [13,32,33,34].

By integrating the PSR into the target volume, the dose to the axillary and interpectoral lymph nodes only modestly increases in the supine position compared to planning on the PTV “B”, since the main tangents have similar angles and the additional dose to the PSR does not result in an increased dose to the axillary lymph nodes. However, the effect of the additional PSR irradiation on the axillary lymph node exposure in the prone position is striking: Compared to planning on the PTV “B”, this significantly increases the dose in the adjacent lymphatic regions since a different field arrangement is needed to cover the PSR. However, the mean dose in the axillary lymph node regions is still significantly lower in the prone position than in the supine position.

### 4.4. Plan Assessments

The OPAF and the individual subfactors analyze which positioning and planning technique is most suitable for which target volume and which performs best in an internal comparison. 

We show for the EBRT of the **PTV “B”** that the mean OPAF for the prone position is significantly higher than for the supine position. The subfactor “homogeneity” reaches higher values in the supine position than in the prone position. The tangential portal arrangements mainly explain this advantage in the supine position: due to the oblique angle, entering rays have a longer path for dose build-up than in the prone position, where the rays hit the breast almost vertically. However, this effect in one subfactor is more than compensated by the subfactors “conformity” and “radiogenic exposure of OAR”, which benefit from the prone position. Looking at the affected sides, the combination “prone position, right breast, and IMRT technique” reaches the highest overall value for the OPAF of 0.75 (SD 0.16). 

In summary—also taking the multivariate analyses into account—the prone position provides the best planning result for the EBRT of the PTV “B”, regardless of the planning technique used or the breast volume.

For the EBRT of the **PTV “B + PSR”,** on the other hand, there is no clear statement about a superior positioning and planning technique concerning the OPAF. 

The subfactors show that radiogenic exposure of OAR benefits from the IMRT technique, but homogeneity clearly benefits from the supine position. The subfactor “conformity” is not significantly higher for the prone position as with EBRT on the PTV “B”; therefore, the OPAF equalizes between supine vs. prone. The reason for this is the different arrangement of the irradiation fields in the prone position. In summary, it can be stated that the EBRT of the PTV “B + PSR” does not show substantial advantages for positioning in the prone position. 

### 4.5. Limitations

In our study, we had to accept several limitations. First, the use of dichotomous plan ratings led to a loss of sensitivity (a plan could only be “good” (“1”) or “not good” (“0”)). We had decided on such an approach because we feared a dilution of the results and no explicit ratings with a more graded ranking. On the other hand, we deliberately refrained from irradiating the supraclavicular lymph nodes. In this way, we wanted to avoid additional irradiation fields that could have blurred the effect of the parasternal irradiation. Ultimately, however, this means that our results on planning for the “B + PSR” show methodological principles but cannot be numerically applied to the clinical situation. In particular, the coirradiation of the supraclavicular lymphatic drainage will result in even higher exposures of the OAR “lung”.

## 5. Conclusions

For the EBRT of the PTV “breast”, positioning in the laterally tilted prone position shows the best planning results regardless of the planning technique.

For the EBRT of the PTV “breast + parasternal region/internal mammary chain”, however, no clinical advantages can be achieved with the techniques used in our study (prone position in free-breathing and step-and-shoot IMRT planning) compared to the classical approach in the supine position. Decisive reductions in the dose exposures of the heart and lungs can only be expected by further developing the prone positioning technique utilizing the “prone crawl device” and EBRT in the deep inspiration breath-hold technique [7]. However, this technique is even more time-consuming and logistically complex than in “simple” positioning in the laterally tilted prone position. Furthermore, the results of proton irradiation [6], which are much better than those of photon irradiation, cannot be used in standard clinical care due to the enormous technical effort and therefore limited availability.

## Figures and Tables

**Table 1:** Exposure of the OARs and ROIs, subdivided according to positioning and planning technique in Gy (mean dose) or percent (V20/V40). Here, only the EBRT of PTV “B” was considered. Exposure of the heart was only calculated for left-sided cancer patients. SD: standard deviation, n.d.: not done.

Exposure of OARs and ROIs, EBRT of PTV “B”	Prone, 3D	Supine, 3D	*p*	Prone, IMRT	Supine, IMRT	*p*
heart, mean dose (left) (Gy)	2.8 (SD 1.02)	4.76 (SD 2.18)	<0.001	3.36 (SD 0.56)	4.16 (SD 1.3)	<0.001
heart, V40 (left) (%)	1.14 (SD 1.2)	4.4 (SD 3.12)	<0.001	0.34 (SD 0.44)	2.87 (SD 1.7)	<0.001
heart, mean dose (right) (Gy)	0.35 (SD 0.07)	0.38 (SD 0.06)	0.98	0.39 (SD 0.1)	0.44 (SD 0.11)	0.87
heart, V40 (right) (%)	0	0	n.d.	0	0	n.d.
ipsilateral lung (left), mean dose (Gy)	1.14 (SD 0.58)	7.83 (SD 1.49)	<0.001	2.49 (SD 0.47)	7.68 (SD 1.28)	<0.001
ipsilateral lung (left), V20 (%)	0.9 (SD 1.03)	14.4 (SD 2.82)	<0.001	0.8 (SD 0.68)	14.84 (SD 2.66)	<0.001
ipsilateral lung (right), mean dose (Gy)	2.33 (SD 1.08)	8.28 (SD 1.62)	<0.001	3.16 (SD 0.78)	8.32 (SD 1.26)	<0.001
ipsilateral lung (right), V20 (%)	3.07 (SD 2.33)	15.64 (SD 3.62)	<0.001	2.43 (SD 1.44)	15.91 (SD 3.09)	<0.001
axillary level I, mean dose (Gy)	13.23 (SD 8.65)	33.01 (SD 10.25)	<0.001	16.6 (SD 6.87)	33.57 (SD 9.46)	<0.001
axillary level I, V40 (%)	17.11 (SD 15.59)	56.05 (SD 22.64)	<0.001	14.35 (SD 11.46)	53.27 (SD 22.14)	<0.001
axillary level II, mean dose (Gy)	1.75 (SD 3.16)	13.01 (SD 12.03)	<0.001	4.55 (SD 3.26)	14.55 (SD 12.08)	<0.001
axillary level II, V40 (%)	0.52 (SD 0.19)	13.73 (SD 20.39)	<0.001	0.32 (SD 2.03)	13.84 (SD 19.97)	<0.001
axillary level III, mean dose (Gy)	1.45 (SD 1.65)	10.17 (SD 10.97)	<0.001	4.12 (SD 3.13)	12.08 (SD 11.57)	<0.001
axillary level III, V40 (%)	0.02 (SD 0.19)	7.57 (SD 16.93)	<0.001	0.01 (SD 0.03)	9.23 (SD 17.18)	<0.001
interpectoral nodes, mean dose (Gy)	15.75 (SD 10.78)	35.57 (SD 11.19)	<0.001	19.95 (SD 8.81)	35.96 (SD 11.56)	<0.001
interpectoral nodes, V40 (%)	20.3 (SD 22.05)	67.12 (SD 23.92)	<0.001	18.42 (SD 17.52)	69.68 (SD 22.92)	<0.001
parasternal region, mean dose (Gy)	9.67 (SD 7.11)	16.49 (SD 10.37)	<0.001	11.8 (SD 6.18)	17.32 (SD 9.37)	<0.001
parasternal region, V40 (%)	4.81 (SD 8.51)	14.49 (SD 22)	<0.001	2.61 (SD 6.16)	11.45 (SD 17.86)	<0.001

**Table 2:** Plan assessment factors for EBRT of PTV “B”. Listed are the overall plan assessment factors by the collective (left and right) as well as the individual subfactors, homogeneity, conformity, and radiogenic exposure of OAR, in each case for positioning in the prone and the supine positions as well as the 3D- and IMRT-planning technique. Each result reaches a value between 0 and 1 (whereby plan quality increases with the value). OPAF: overall plan assessment factor, OAR: organs at risk, SD: standard deviation.

Plan Assessment Factors, EBRT of PTV “B”	Prone, 3D	Supine, 3D	*p*	Prone, IMRT	Supine, IMRT	*p*
OPAF, whole collective	0.61 (SD 0.1)	0.42 (SD 0.26)	<0.001	0.69 (SD 0.18)	0.45 (SD 0.15)	<0.001
OPAF, left breast	0.6 (SD 0.11)	0.45 (SD 0.26)	<0.001	0.62 (SD 0.18)	0.48 (SD 0.19	<0.001
OPAF, right breast	0.63 (SD 0.07)	0.38 (SD 0.27)	<0.001	0.75 (SD 0.16)	0.43 (SD 0.11)	<0.001
subfactor “homogeneity”	0.03 (SD 0.16)	0.78 (SD 0.42)	<0.001	0.3 (SD 0.46)	0.98 (SD 0.16)	<0.001
subfactor “conformity”	0.85 (SD 0.26)	0.4 (SD 0.46)	<0.001	0.78 (SD 0.25)	0.33 (SD 0.38)	<0.001
subfactor “radiogenic exposure of OAR”	0.96 (SD 0.13)	0.08 (SD 0.17)	<0.001	0.98 (SD 0.07)	0.06 (SD 0.16)	<0.001

**Table 3:** Exposure of the OARs and ROIs, subdivided according to positioning and planning technique in Gy (mean dose) or percent (V20/V40). Here, only EBRT of PTV “B + PSR” was considered. SD: standard deviation.

Exposure of OARs and ROIs, EBRT of PTV “B + PSR”	Prone, 3D	Supine, 3D	*p*	Prone, IMRT	Supine, IMRT	*p*
heart, mean dose (left) (Gy)	7.24 (SD 2.23)	7.3 (SD 2.9)	4.7	7.35 (SD 1.78)	6 (SD 2.02)	0.015
heart, V40 (left) (%)	7.19 (SD 3.39)	7.87 (SD 4.54)	0.3	2.53 (SD 1.53)	3.77 (SD 2.41)	0.03
heart, mean dose (right) (Gy)	1.8 (SD 0.82)	1.07 (SD 0.42)	<0.001	3.54 (SD 1.19)	1.61 (SD 0.94)	<0.001
heart, V40 (right) (%)	0.52 (SD 0.83)	0.04 (SD 0.15)	0.008	0.3 (SD 0.52)	0.13 (SD 0.58)	0.04
ipsilateral lung (left), mean dose (Gy)	7.42 (SD 2.53)	13.12 (SD 2.6)	<0.001	7.52 (SD 1.72)	12.65 (SD 0.99)	<0.001
ipsilateral lung (left), V20 (%)	14.44 (SD 5.38)	27.61 (SD 4.45)	<0.001	11.72 (SD 3.98)	24.78 (SD 2.45)	<0.001
ipsilateral lung (right), mean dose (Gy)	9.76 (SD 2.82)	14.34 (SD 2.55)	<0.001	9.52 (SD 1.78)	14.06 (SD 2.01)	<0.001
ipsilateral lung (right), V20 (%)	18.77 (SD 7.59)	30.44 (SD 6.22)	<0.001	15.21 (SD 5.62)	28.51 (SD 5.02)	<0.001
axillary level I, mean dose (Gy)	31.8 (SD 8.58)	35.95 (SD 8.19)	0.015	26.79 (SD 6.76)	33.57 (SD 9.46)	<0.001
axillary level I, V40 (%)	51.11 (SD 20.47)	62.05 (SD 18.99)	0.007	24.4 (SD 16.94)	61.3 (SD 17.97)	<0.001
axillary level II, mean dose (Gy)	8.21 (SD 7.32)	13.03 (SD 10.58)	0.01	10.53 (SD 6.86)	16.14 (SD 11.09)	0.004
axillary level II, V40 (%)	3.68 (SD 12.22)	11.32 (SD 18.02)	0.02	1.94 (SD 6.32)	12.96 (SD 17.2)	<0.001
axillary level III, mean dose (Gy)	6.74 (SD 6.67)	10.96 (SD 9.05)	0.01	9 (SD 5.98)	14.32 (SD 10.76)	0.04
axillary level III, V40 (%)	1.74 (SD 8.56)	6.39 (SD 13.32)	0.07	0.32 (SD 1.26)	8.37 (SD 16.16)	0.001
interpectoral nodes, mean dose (Gy)	26.2 (SD 10.43)	35.53 (SD 11.14)	<0.001	24.6 (SD 7.82)	36.76 (SD 10.84)	<0.001
interpectoral nodes, V40 (%)	40.45 (SD 23.99)	66.77 (SD 23.82)	<0.001	23.31 (SD 19.11)	69.33 (SD 23.1)	<0.001

**Table 4:** Plan assessment factors for EBRT of PTV “B-PSR”. Listed are the overall plan assessment factors by the collective (left and right) as well as the individual subfactors, homogeneity, conformity, and radiogenic exposure of OAR, in each case for positioning in the prone and the supine positions as well as the 3D- and IMRT-planning techniques. Each result reaches a value between 0 and 1 (whereby plan quality increases with the value). OPAF: overall plan assessment factor; OAR: organs at risk; SD: standard deviation.

Plan Assessment Factors, EBRT of PTV “B + PSR”	Prone, 3D	Supine, 3D	*p*	Prone, IMRT	Supine, IMRT	*p*
OPAF, whole collective	0.47 (SD 0.18)	0.51 (SD 0.2)	0.13	0.59 (SD 0.17)	0.58 (SD 0.22)	0.22
OPAF, left breast	0.46 (SD 0.18)	0.49 (SD 0.23)	0.24	0.6 (SD 0.17)	0.54 (SD 0.22)	0.43
OPAF, right breast	0.48 (SD 0.18)	0.54 (SD 0.17)	0.21	0.57 (SD 0.18)	0.61 (SD 0.22)	0.3
subfactor “homogeneity”	0.35 (SD 0.26)	0.56 (SD 0.23)	<0.001	0.44 (SD 0.32)	0.61 (SD 0.33)	0.009
subfactor “conformity”	0.41 (SD 0.36)	0.56 (SD 0.41)	0.042	0.59 (SD 0.25)	0.69 (SD 0.39)	0.12
subfactor “radiogenic exposure of OAR”	0.64 (SD 0.31)	0.42 (SD 0.3)	0.001	0.73 (SD 0.19)	0.43 (SD 0.23)	<0.001

**Table 5:** Overview of the results. “+” means a significant advantage compared to the other planning techniques or patient positioning; “++” marks additionally a clear clinical advantage.

	Prone, 3D	Prone, IMRT	Supine, 3D	Supine, IMRT
**PTV “B”**				
heart	**+**	**+**		
ipsilateral lung	**+**	**+**		
ipsilateral lung	**+**	**+**		
axillary and other lymphatic regions	**+**	**+**		
*plan assessment*				
– homogeneity		**+**	**++**	**++**
– conformity	**+**	**+**		
– radiogenic exposure of OAR	**++**	**++**		
– overall plan assessment	**+**	**+**		
**PTV “B + PSR”**				
heart, left-breast EBRT				**+**
heart, right-breast EBRT			**+**	**+**
ipsilateral lung	**++**	**++**		
axillary and other lymphatic regions	**+**	**+**		
*plan assessment*				
– homogeneity			**++**	**++**
– conformity		**+**	**+**	**+**
– radiogenic exposure of OAR	**+**	**+**		
– overall plan assessment				

## Data Availability

Pseudonymized data are available on request from the corresponding author.

## Supplementary Materials

The following supporting information can be downloaded at: https://www.mdpi.com/article/10.3390/jpm12040653/s1. Tables S1–S4 and Figures S1–S18 are shown in the Supplementary Materials.
