# Supplementary material for: Radiotherapy of Breast Cancer in Laterally Tilted Prone vs. Supine Position: What about the Internal Mammary Chain?
Source: J Pers Med. 2022 Apr 18;12(4):653. doi: 10.3390/jpm12040653 (PMC9031123; doi:10.3390/jpm12040653)
Supplement: Supplementary file 1 [file jpm-12-00653-s001.zip › jpm-1647370(2).docx]

Supplementarry Material

Temme, N.; Hermann, R.M.; Hinsche, T.; Becker, J.N.; Sonnhoff, M.; Kaltenborn, A.; Carl, U.M.; Christiansen, H.; Nitsche, M. Radiotherapy of breast cancer in laterally tilted prone vs. supine position: what about the internal mammary chain?

Content

[table S1: patient characteristics with left-sided tumors 2](#_Toc97037701)

[table S2: patient characteristics with right-sided tumors 3](#_Toc97037702)

[table S3: multivariable logistic regression for plan quality EBRT of “PTV-B” 4](#_Toc97037703)

[table S4: multivariable logistic regression for plan quality EBRT of “PTV-B+PSR” 5](#_Toc97037704)

[figure S1: mean dose OAR "heart", left-sided tumors, EBRT of “PTV-B” 6](#_Toc97037705)

[figure S2: V40 OAR "heart", left-sided tumors, EBRT of “PTV-B” 7](#_Toc97037706)

[figure S3: mean dose OAR "ipsilateral lung", EBRT of “PTV-B” 8](#_Toc97037707)

[figure S4: mean dose ROI "axillary level I lymph nodes", EBRT of “PTV-B” 9](#_Toc97037708)

[figure S5: V40 ROI "axillary level I lymph nodes", EBRT of “PTV-B” 10](#_Toc97037709)

[figure S6: mean dose ROI "axillary level II lymph nodes", EBRT of “PTV-B” 11](#_Toc97037710)

[figure S7: mean dose ROI "axillary level III lymph nodes", EBRT of “PTV-B” 12](#_Toc97037711)

[figure S8: mean dose ROI "Rotter lymph nodes", EBRT of “PTV-B” 13](#_Toc97037712)

[figure S9: mean dose ROI "internal mammary chain", EBRT of “PTV-B” 14](#_Toc97037713)

[figure S10: mean dose OAR "heart", left-sided tumors, EBRT of “PTV-B+PSR” 15](#_Toc97037714)

[figure S11: V40 OAR "heart", left-sided tumors, EBRT of “PTV-B+PSR” 16](#_Toc97037715)

[figure S12: mean dose OAR "heart", right-sided tumors, EBRT of “PTV-B+PSR” 17](#_Toc97037716)

[figure S13: mean dose OAR "ipsilateral lung", EBRT of “PTV-B+PSR” 18](#_Toc97037717)

[figure S14: mean dose ROI "axillary level I lymph nodes", EBRT of “PTV-B+PSR” 19](#_Toc97037718)

[figure S15: V40 ROI "axillary level I lymph nodes", EBRT of “PTV-B+PSR” 20](#_Toc97037719)

[figure S16: mean dose ROI "axillary level II lymph nodes", EBRT of “PTV-B+PSR” 21](#_Toc97037720)

[figure S17: mean dose ROI "axillary level III lymph nodes", EBRT of “PTV-B+PSR” 22](#_Toc97037721)

[figure S18: mean dose ROI "Rotter lymph nodes", EBRT of “PTV-B+PSR” 23](#_Toc97037722)

# table S1: patient characteristics with left-sided tumors

Left-sided irradiated patients included in the study. The age is given in years, the length in meters, the weight in kilograms, and the PTV "B" volume in cm³ (as shown in the planning system Pinnacle³).

| **patients with tumors in the left breast** | | | | | | |
| --- | --- | --- | --- | --- | --- | --- |
|  | age | length | weight | BMI | volume  PTV „B“  (supine) | Volume  PTV „B“ (prone) |
| 1 | 29 | 1,7 | 68 | 23,5 | 609 | 652 |
| 2 | 51 | 1,7 | 77 | 26,4 | 1268 | 1084 |
| 3 | 77 | 1,76 | 72 | 23,2 | 936 | 776 |
| 4 | 65 | 1,68 | 65 | 23,0 | 783 | 740 |
| 5 | 53 | 1,68 | 75 | 26,6 | 692 | 491 |
| 6 | 55 | 1,7 | 80 | 27,7 | 936 | 853 |
| 7 | 50 | 1,62 | 63 | 24 | 786 | 730 |
| 8 | 42 | 1,59 | 54 | 21,4 | 471 | 423 |
| 9 | 66 | 1,66 | 62 | 22,5 | 884 | 772 |
| 10 | 70 | 1,62 | 62 | 23,6 | 406 | 499 |
| 11 | 52 | 1,75 | 78 | 25,5 | 291 | 316 |
| 12 | 67 | 1,68 | 69 | 24,4 | 665 | 958 |
| 13 | 62 | 1,7 | 77 | 26,6 | 684 | 740 |
| 14 | 53 | 1,73 | 65 | 21,7 | 466 | 458 |
| 15 | 51 | 1,74 | 78 | 25,7 | 736 | 778 |
| 16 | 75 | 1,69 | 54 | 18,9 | 509 | 513 |
| 17 | 65 | 1,65 | 68 | 25,0 | 637 | 616 |
| 18 | 50 | 1,65 | 78 | 28,7 | 1092 | 1177 |
| 19 | 50 | 1,65 | 52 | 19,1 | 620 | 569 |
| 20 | 58 | 1,78 | 83 | 26,2 | 1211 | 1170 |

# table S2: patient characteristics with right-sided tumors

Right-sided irradiated patients included in the study. The age is given in years, the length in meters, the weight in kilograms, and the PTV "B" volume in cm³ (as shown in the planning system Pinnacle³).

| **patients with tumors in the right breast** | | | | | | |
| --- | --- | --- | --- | --- | --- | --- |
|  | age | length | weight | BMI | volume  PTV „B“ (supine) | Volume  PTV B  (prone) |
| **1** | 48 | 1,68 | 56 | 19,8 | 528 | 489 |
| **2** | 84 | 1,58 | 48 | 19,2 | 179 | 204 |
| **3** | 55 | 1,72 | 74 | 25,1 | 772 | 939 |
| **4** | 57 | 1,67 | 67 | 24,3 | 575 | 675 |
| **5** | 51 | 1,68 | 75 | 26,6 | 688 | 617 |
| **6** | 68 | 1,56 | 61 | 25,1 | 1399 | 1295 |
| **7** | 63 | 1,67 | 68 | 24,4 | 1286 | 1179 |
| **8** | 76 | 1,68 | 73 | 25,9 | 637 | 569 |
| **9** | 34 | 1,7 | 64 | 22,1 | 348 | 315 |
| **10** | 55 | 1,81 | 64 | 19,5 | 666 | 666 |
| **11** | 53 | 1,7 | 80 | 27,7 | 844 | 936 |
| **12** | 48 | 1,78 | 68 | 21,5 | 679 | 735 |
| **13** | 43 | 1,64 | 65 | 24,2 | 666 | 669 |
| **14** | 54 | 1,7 | 65 | 22,5 | 674 | 714 |
| **15** | 52 | 1,65 | 67 | 24,6 | 684 | 756 |
| **16** | 69 | 1,58 | 57 | 22,8 | 900 | 911 |
| **17** | 34 | 1,72 | 78 | 26,4 | 841 | 915 |
| **18** | 58 | 1,58 | 65 | 26,0 | 1314 | 1313 |
| **19** | 49 | 1,7 | 85 | 29,4 | 1419 | 1520 |
| **20** | 34 | 1,67 | 75 | 26,9 | 858 | 839 |

# table S3: multivariable logistic regression for plan quality EBRT of “PTV-B”

Possible relevant factors (prone vs. supine; 3D vs. IMRT; the volume of PTV “Breast” < 600 cm³ vs. 600 – 900cm³ vs. > 900 cm³) on planning quality (subfactors “homogeneity”, “conformity”, and “radiogenic exposure of OAR” were included in multivariate regression analyses. p-value <0.05 was defined as statistically significant (italic type)

| **PTV”B”** | **factor** | **odds ratio** | **CI 95%** | **P** |
| --- | --- | --- | --- | --- |
| “homogeneity” |  |  |  |  |
|  | BMI | -0.001 | -0021 - 0.019 | 0.913 |
|  | PTV < 600 cm³ | -0.021 | -0.141 - 0.098 | 0.727 |
|  | PTV > 900 cm³ | 0.008 | -0.108 - 0.125 | 0.138 |
|  | *prone position* | *-0.194* | *-0.285 - -0.103* | *<0.001* |
|  | 3D / IMRT | -0.069 | -0.16 - 0.022 | 0.137 |
|  |  |  |  |  |
| “conformity” |  |  |  |  |
|  | *BMI* | *-0.023* | *-0.046 – 0* | *0.048* |
|  | PTV < 600 cm³ | 0.106 | -0.03 – 0.242 | 0.125 |
|  | PTV > 900 cm³ | -0.092 | -0.224 – 0.041 | 0.173 |
|  | *prone position* | *0.45* | *0.347 – 0.553* | *< 0.001* |
|  | 3D / IMRT | 0.075 | -0.028 – 0.178 | 0.154 |
|  |  |  |  |  |
| “OAR exposure” |  |  |  |  |
|  | BMI | 0.0 | -0.146 – 0.33 | 0.927 |
|  | PTV < 600 cm³ | -0.007 | -0.065 – 0.05 | 0.804 |
|  | PTV > 900cm³ | -0.03 | -0.086 – 0.025 | 0.283 |
|  | *prone position* | *0.9* | *0.856 – 0.944* | *< 0.001* |
|  | 3D / IMRT | 0.0 | -0.044 – 0.044 | 1.0 |

# table S4: multivariable logistic regression for plan quality EBRT of “PTV-B+PSR”

Possible relevant factors (prone vs. supine; 3D vs. IMRT; the volume of PTV “Breast” < 600 cm³ vs. 600 – 900cm³ vs. > 900 cm³) on planning quality (subfactors “homogeneity”, “conformity”, and “radiogenic exposure of OAR” were included in multivariate regression analyses. p-value <0.05 was defined as statistically significant (italic type)

| **PTV”B+PSR”** | **factor** | **odds ratio** | **CI 95%** | **p** |
| --- | --- | --- | --- | --- |
| “homogeneity” |  |  |  |  |
|  | BMI | -0.041 | -0.063 - -0.018 | < 0.001 |
|  | PTV < 600 cm³ | 0.112 | -0.021 – 0.244 | 0.098 |
|  | PTV > 900 cm³ | -0.08 | -0.209 – 0.049 | 0.221 |
|  | *prone position* | *-0.125* | *-0.226 - -0.024* | *0.0015* |
|  | *3D* | *-0.15* | *-0.251 - -0.049* | *0.004* |
|  |  |  |  |  |
| “conformity” |  |  |  |  |
|  | BMI | -0.01 | -0.021 – 0.019 | 0.913 |
|  | PTV < 600 cm³ | -0.021 | -0.141 – 0.098 | 0.727 |
|  | PTV > 900 cm³ | 0.008 | -0.108 – 0.125 | 0.89 |
|  | *prone position* | *-0.194* | *-0.285 - -0.103* | *< 0.001* |
|  | 3D / IMRT | -0.069 | -0.16 – 0.022 | 0.137 |
|  |  |  |  |  |
| “OAR exposure” |  |  |  |  |
|  | *BMI* | *-0.026* | *-0.044 - -0.008* | *0.005* |
|  | PTV < 600 cm³ | -0.047 | -0.153 – 0.059 | 0.382 |
|  | PTV > 900cm³ | -0.015 | -0.116 – 0.089 | 0.776 |
|  | *prone position* | *0.263* | *0.182 – 0.343* | *< 0.001* |
|  | 3D / IMRT | -0.05 | -0.131 – 0.031 | 0.223 |

# figure S1: mean dose OAR "heart", left-sided tumors, EBRT of “PTV-B”


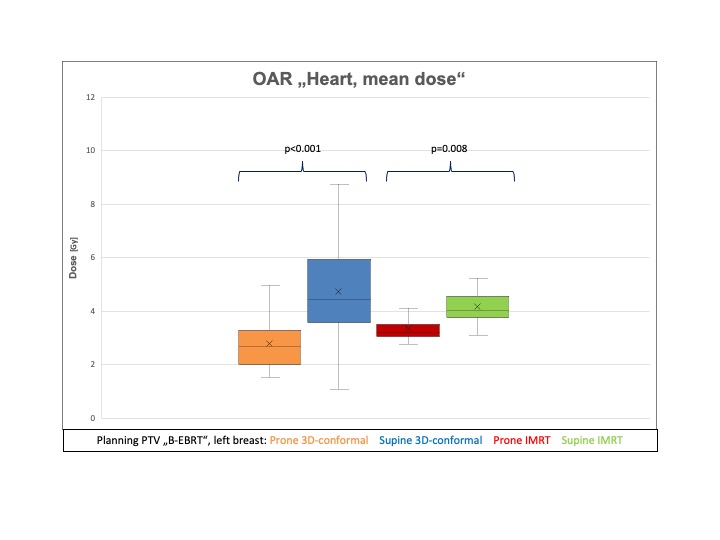


Representation of the mean dose of the organ at risk "heart" in the box plot for EBRT of left-sided patients (n = 20), in each case for positioning in the prone position and supine position as well as for the 3D EBRT and IMRT EBRT technique on the PTV "B". The significance brackets indicate the statistical significance between the patient positioning "supine vs. prone". The upper and lower quartiles are each shown with a vertical line (maximum 1.5 times the interquartile range), the middle quartiles are each shown with an area. Outliers are not given. There is a horizontal line between the middle quartiles indicating the median value. The mean value is marked with an "x".

Values are given in table 2: mean heart dose in the prone position with 3D EBRT calculated 2.8 Gy (SD 1.02) vs. 4.76 Gy (SD 2.18) in the supine position, and 3.36 Gy (SD 0.56) / 4.16 Gy (SD 1.3) with IMRT EBRT.

# figure S2: V40 OAR "heart", left-sided tumors, EBRT of “PTV-B”


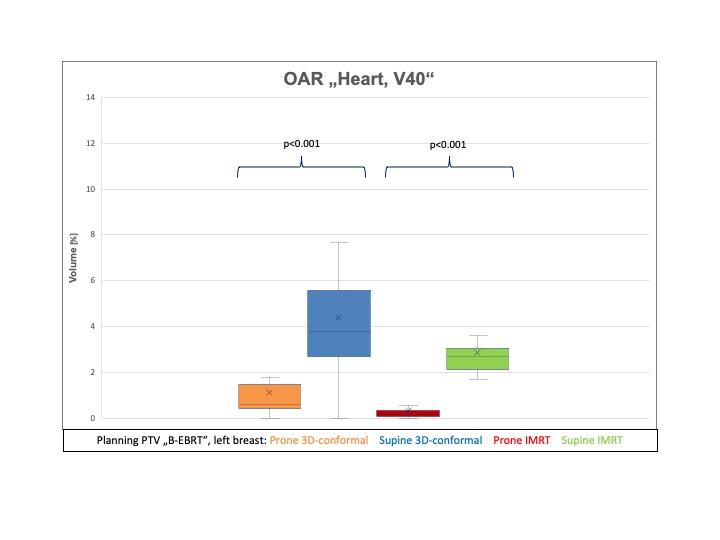
 For details on the method of presentation, see the capture for figure S1. The values are given for the collective of patients irradiated on the left side (n=20). The heart volume exposed to a dose of at least 40 Gy (V40) was 1.14 % (SD 1.2 %) in mean in the prone position for 3D EBRT and 0.34 % (SD 0.44 %) for IMRT EBRT. In the supine position, the V40 value was significantly higher both for 3D at 4.4 % (SD 3.12 %) and for IMRT at 2.87 % (SD 1.7 %), p<0.001 for each technique.

IMRT EBRT in comparison to 3D EBRT significantly reduced V40 values but not independent of patient positioning: IMRT EBRT in the supine position was not significantly better than 3D EBRT in the prone position.

# figure S3: mean dose OAR "ipsilateral lung", EBRT of “PTV-B”


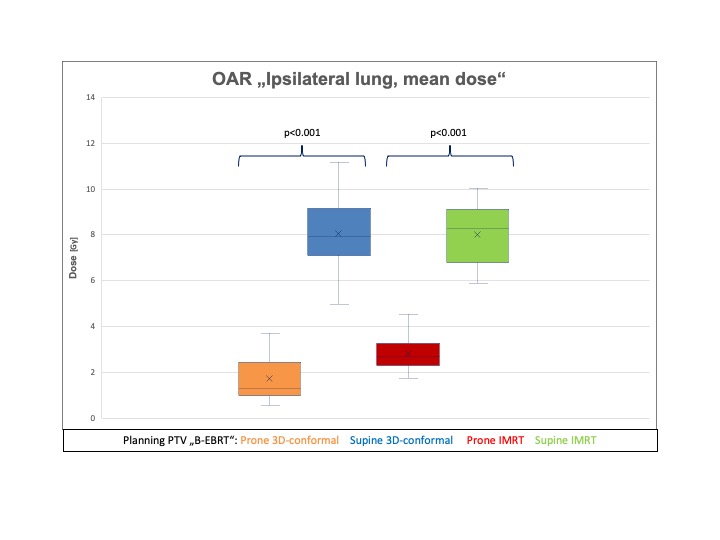


For details on the method of presentation, see the caption for figure S1. The values are given for all patients (n=40). The supine position significantly reduced the mean dose to the ipsilateral lung in comparison to the prone position: for 3D EBRT from 8.06 Gy (SD 1.55) to 1.74 Gy (SD 1.05), for IMRT EBRT from 8.0 Gy SD (1.3) to 2.81 Gy (SD 0.72).

# figure S4: mean dose ROI "axillary level I lymph nodes", EBRT of “PTV-B”


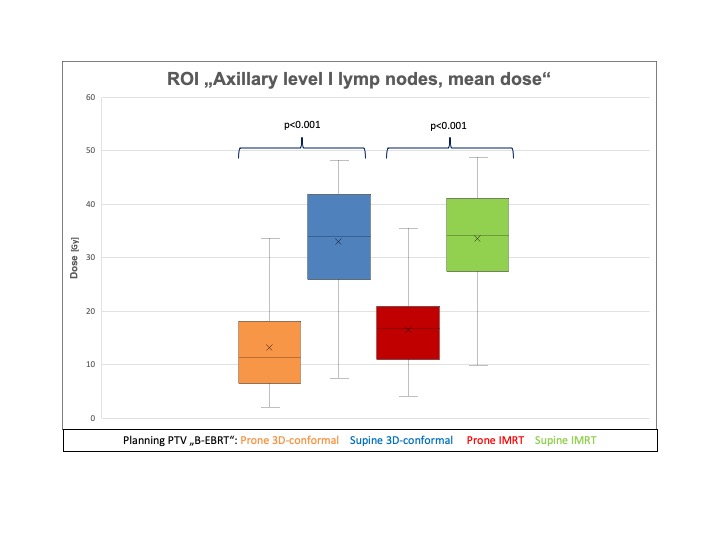


For details on the method of presentation, see the caption for figure S1. The values are given for all patients (n=40). The mean dose in the axillary level I lymphatic region was significantly lower in the prone position compared to the supine position (p < 0.01 for each planning technique): prone 3D EBRT calculated a mean dose of 13.23 Gy (SD 8.65 Gy), IMRT EBRT 16.6 Gy (SD 6.87 Gy). In the supine position, 3D EBRT resulted in 33.01 Gy (SD 10.25 Gy), and IMRT EBRT in 33.57 Gy (SD 9.46 Gy).

# figure S5: V40 ROI "axillary level I lymph nodes", EBRT of “PTV-B”


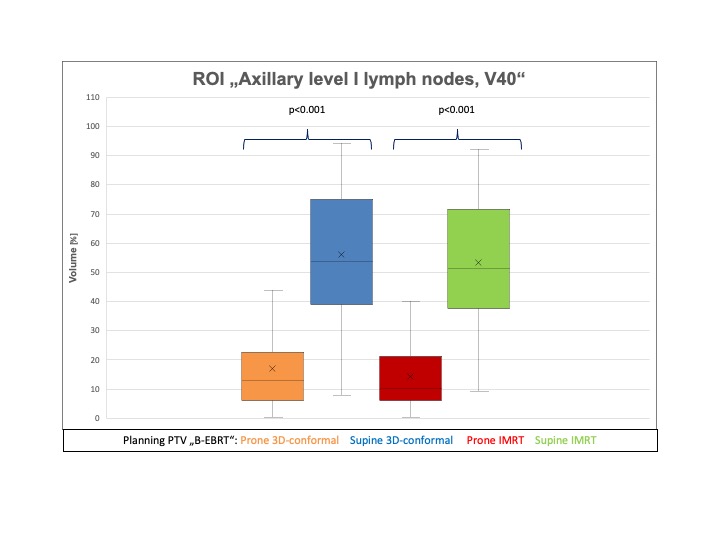


For details on the method of presentation, see the caption for figure S1. The values are given for all patients (n=40). In the prone position, the mean V40-values in the axillary level I lymphatic region was 17.11% (SD 15.59 %, 3D EBRT ) and 14.35 % (SD 11.46 %, IMRT EBRT). In the supine position, the V40-values were significantly higher with 56.05 % (SD 22.64 %) for 3D EBRT, respectively 53.27 % (SD 22.14 %) for IMRT EBRT.

# figure S6: mean dose ROI "axillary level II lymph nodes", EBRT of “PTV-B”


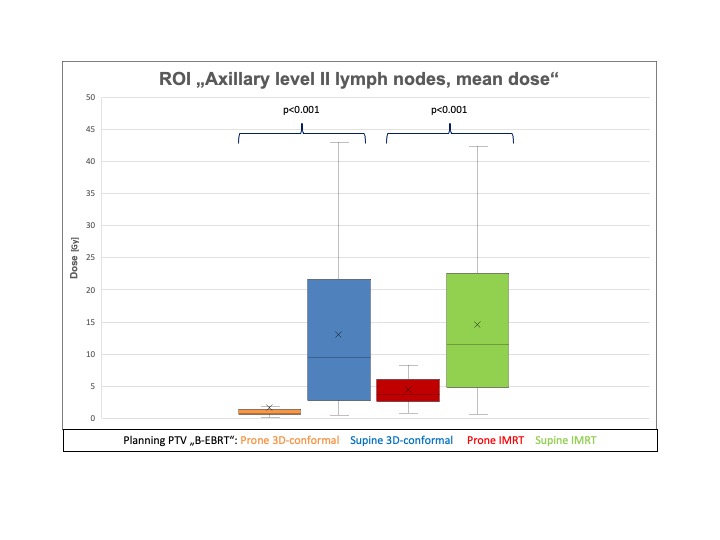


For details on the method of presentation, see the caption for figure S1. The values are given for all patients (n=40). In the prone position, the mean dose in the axillary level II lymphatic region was 1.75 Gy (SD 3.16 Gy, 3D EBRT ) and 4.55 Gy (SD 3.26 Gy, IMRT EBRT). In the supine position, the mean doses were significantly higher with 13.01 Gy (SD 12.03 Gy) for 3D EBRT and 14.55 Gy (SD 12.08 Gy) for IMRT EBRT.

# figure S7: mean dose ROI "axillary level III lymph nodes", EBRT of “PTV-B”


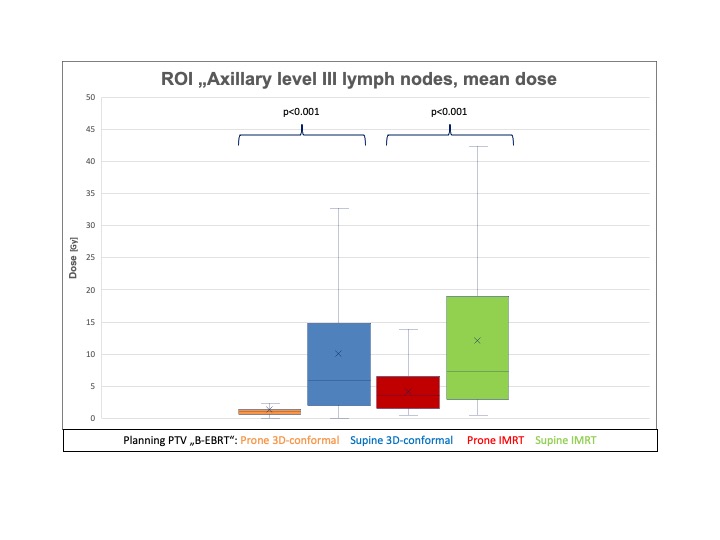


For details on the method of presentation, see the caption for figure S1. The values are given for all patients (n=40). In the prone position, the mean dose in the axillary level III lymphatic region was 1.45 Gy (SD 1.65 Gy, 3D EBRT ) and 4.12 Gy (SD 3.13 Gy, IMRT EBRT). In the supine position, the mean doses were significantly higher with 10.17 Gy (SD 10.97 Gy) for 3D EBRT and 12.08 Gy (SD 11.57 Gy) for IMRT EBRT.

# figure S8: mean dose ROI "Rotter lymph nodes", EBRT of “PTV-B”


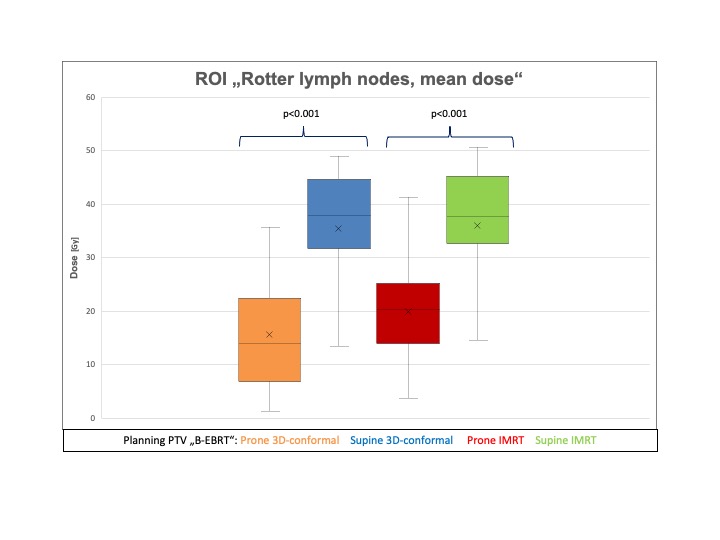


For details on the method of presentation, see the caption for figure S1. The values are given for all patients (n=40). In the prone position, the mean dose in the “Rotter” lymphatic region was 15.75 Gy (SD 10.78 Gy, 3D EBRT ) and 19.95 Gy (SD 8.81 Gy, IMRT EBRT). In the supine position, the mean doses were significantly higher with 35.57 Gy (SD 11.19 Gy) for 3D EBRT and 35.96 Gy (SD 11.56 Gy) for IMRT EBRT.

# figure S9: mean dose ROI "internal mammary chain", EBRT of “PTV-B”


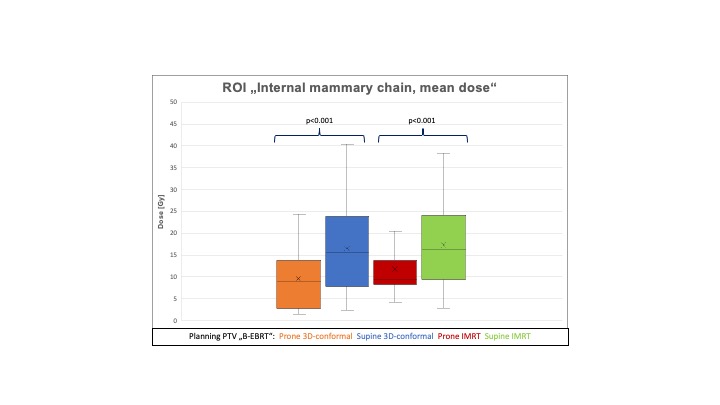


For details on the method of presentation, see the caption for figure S1. The values are given for all patients (n=40). In the prone position, the mean dose in the parasternal lymphatic region (“internal mammary chain”) was 9.67 Gy (SD 7.11 Gy, 3D EBRT) and 11.8 Gy (SD 6.18 Gy, IMRT EBRT). In the supine position, the mean doses were significantly higher with 16.49 Gy (SD 10.37 Gy) for 3D EBRT and 17.32 Gy (SD 9.37 Gy) for IMRT EBRT.

# figure S10: mean dose OAR "heart", left-sided tumors, EBRT of “PTV-B+PSR”


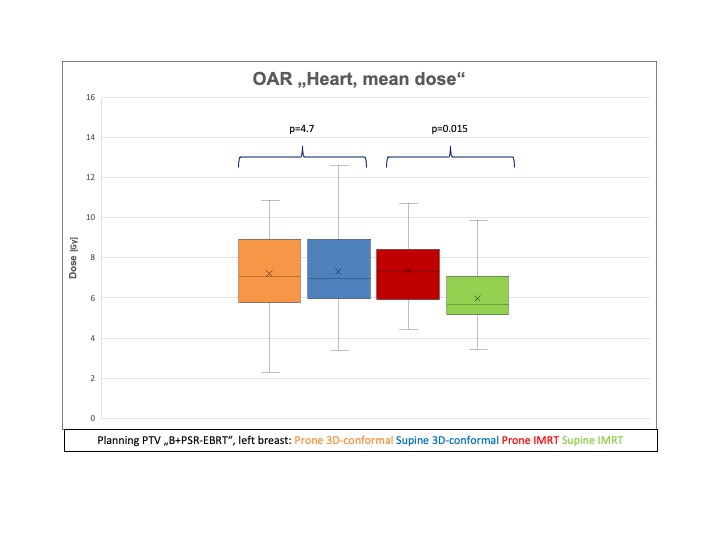


Representation of the mean dose of the organ at risk "heart" in the box plot for EBRT of left-sided patients (n = 20), in each case for positioning in the prone position and supine position as well as for the 3D EBRT and IMRT EBRT technique on the PTV "B-PSR". The significance brackets indicate the statistical significance between the patient positioning "supine vs. prone". The upper and lower quartiles are each shown with a vertical line (maximum 1.5 times the interquartile range), the middle quartiles are each shown with an area. Outliers are not given. There is a horizontal line between the middle quartiles indicating the median value. The mean value is marked with an "x".

Values are given in table 4: mean heart dose in the prone position with 3D EBRT calculated 7.24 Gy (SD 2.23) vs. 7.3 Gy (SD 2.9) in the supine position, and 7.35 Gy (SD 1.78) / 6 Gy (SD 2.02) with IMRT EBRT.

# figure S11: V40 OAR "heart", left-sided tumors, EBRT of “PTV-B+PSR”


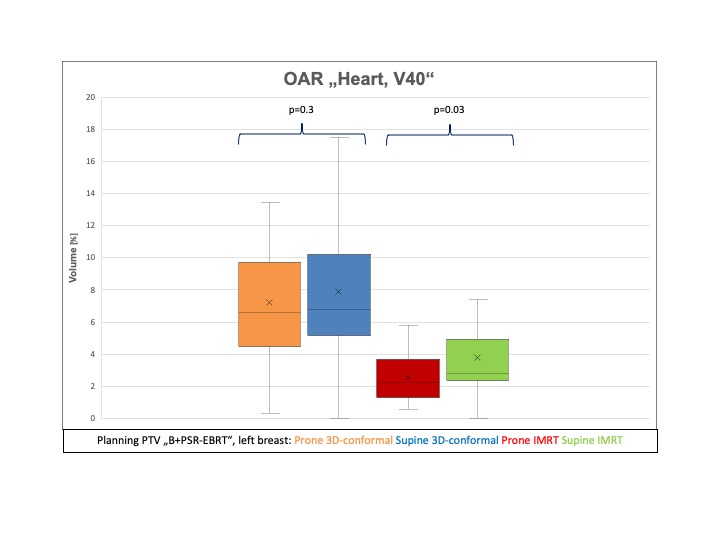
 For details on the method of presentation, see the capture for figure S10. The values are given for the collective of patients irradiated on the left side (n=20). The heart volume exposed to a dose of at least 40 Gy (V40) was 7.19 % (SD 3.39 %) in mean in the prone position for 3D EBRT and 2.53 % (SD 1.53 %) for IMRT EBRT. In the supine position, the V40 value was 7.87% (SD 4.54 %) for 3D EBRT and 3.77% (SD 2.41%) for IMRT EBRT. Thus, prone IMRT significantly reduced high dose exposure of the heart in comparison to supine IMRT (p = 0.03) and 3D EBRT.

# figure S12: mean dose OAR "heart", right-sided tumors, EBRT of “PTV-B+PSR”


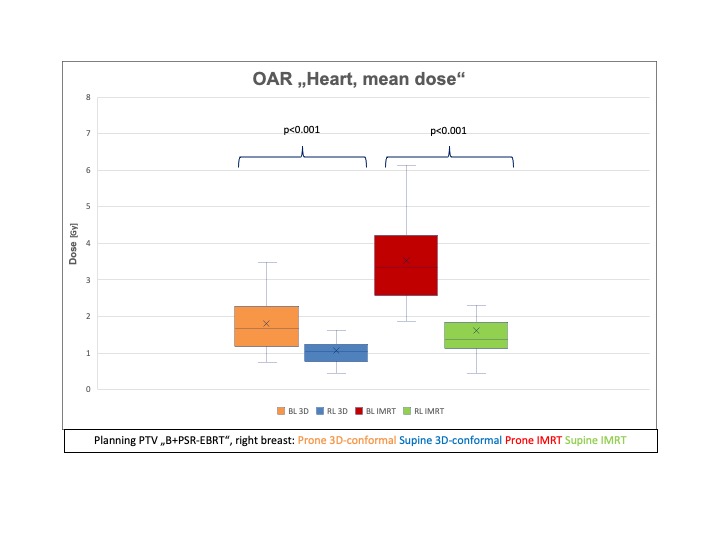


For details on the method of presentation, see the capture for figure S10. The values are given for the collective of patients irradiated on the right side (n=20). The mean heart volume was 1.8 Gy (SD 0.82 Gy) in the prone position for 3D EBRT and 3.54 Gy (SD 1.19 Gy) for IMRT EBRT. In the supine position, the mean dose was 1.07 Gy (SD 0.42 Gy) for 3D EBRT and 1.61 Gy (SD 0.94 Gy) for IMRT EBRT. Thus, the supine position significantly decreased cardiac exposure in comparison to the prone position.

# figure S13: mean dose OAR "ipsilateral lung", EBRT of “PTV-B+PSR”


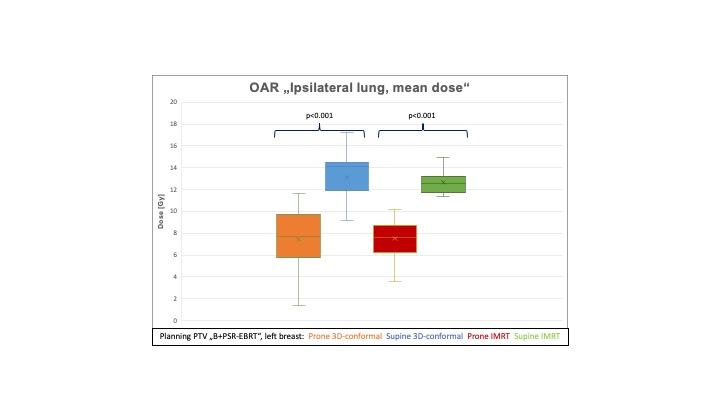


For details on the method of presentation, see the caption for figure S10. The values are given for all patients (n=40). The prone position significantly reduced the mean dose of the ipsilateral lung. In the prone position, 3D EBRT resulted in a mean dose of 8.6 Gy (SD 2.89 Gy) and IMRT EBRT in 8.52 Gy (SD 2 Gy). In the supine position, 3D EBRT calculated a mean dose of 13.73 Gy (SD 2.62 Gy) and IMRT EBRT of 13.36 Gy (SD 1.72 Gy).

# figure S14: mean dose ROI "axillary level I lymph nodes", EBRT of “PTV-B+PSR”


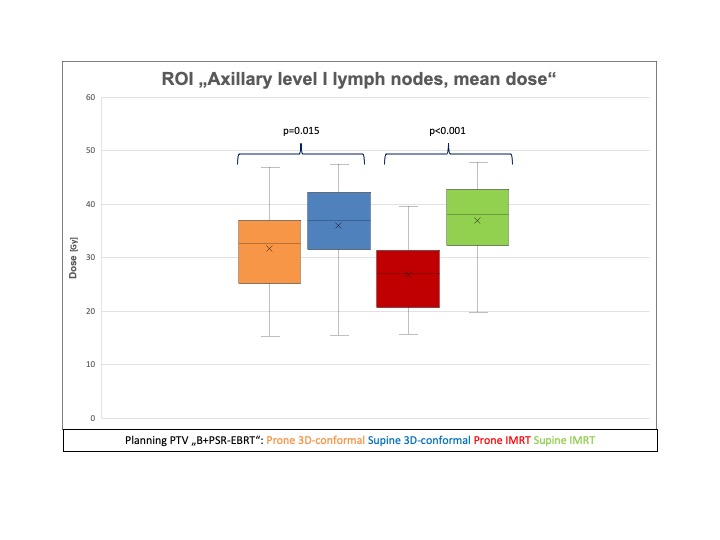


For details on the method of presentation, see the caption for figure S10. The values are given for all patients (n=40). The mean dose in the axillary level I lymphatic region was significantly lower in the prone position compared to the supine position.

In the prone position 3D EBRT calculated a mean dose of 31.8 Gy (SD 8.58 Gy), IMRT EBRT 26.79 Gy (SD 6.76 Gy). In the supine position, 3D EBRT resulted in 35.95 Gy (SD 8.19 Gy), and IMRT EBRT in 33.57 Gy (SD 9.46 Gy).

# figure S15: V40 ROI "axillary level I lymph nodes", EBRT of “PTV-B+PSR”


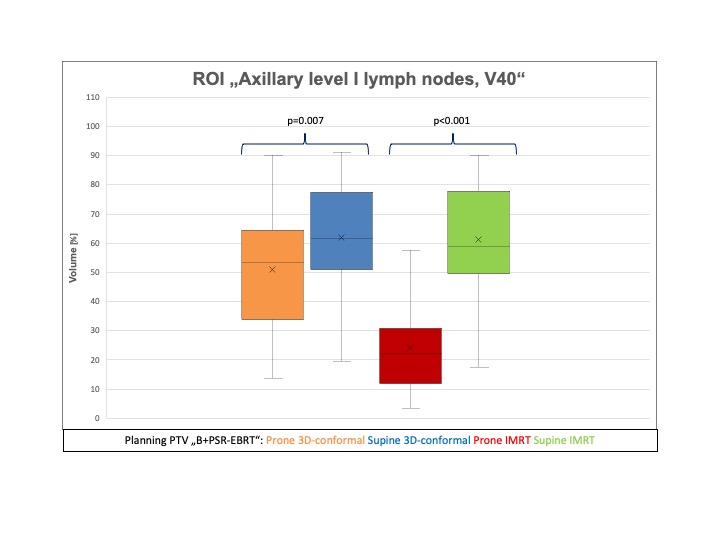


For details on the method of presentation, see the caption for figure S10. The values are given for all patients (n=40). In the prone position, the exposure to high radiation doses was lower than in the supine position.

In the prone position, the mean V40-values in the axillary level I lymphatic region was 51.11% (SD 20.47 %, 3D EBRT ) and just 24.4 % (SD 16.94 %, IMRT EBRT). In the supine position, the V40-values were significantly higher with 62.05 % (SD 18.99 %, p = 0.007) for 3D EBRT, respectively 61.03 % (SD 17.97 %, p< 0.001) for IMRT EBRT.

# figure S16: mean dose ROI "axillary level II lymph nodes", EBRT of “PTV-B+PSR”


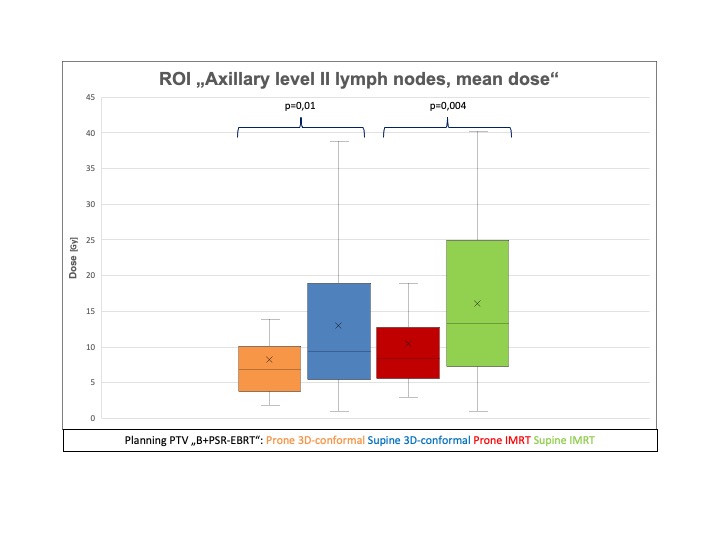


For details on the method of presentation, see the caption for figure S10. The values are given for all patients (n=40). The mean dose in the axillary level II lymphatic region was significantly lower in the prone position compared to the supine position.

In the prone position, 3D EBRT calculated a mean dose of 8.21 Gy (SD 7.32 Gy), IMRT EBRT 10.53 Gy (SD 6.86 Gy). In the supine position, 3D EBRT resulted in 13.03 Gy (SD 10.58 Gy), and IMRT EBRT in 16.14 Gy (11.09 Gy).

# figure S17: mean dose ROI "axillary level III lymph nodes", EBRT of “PTV-B+PSR”


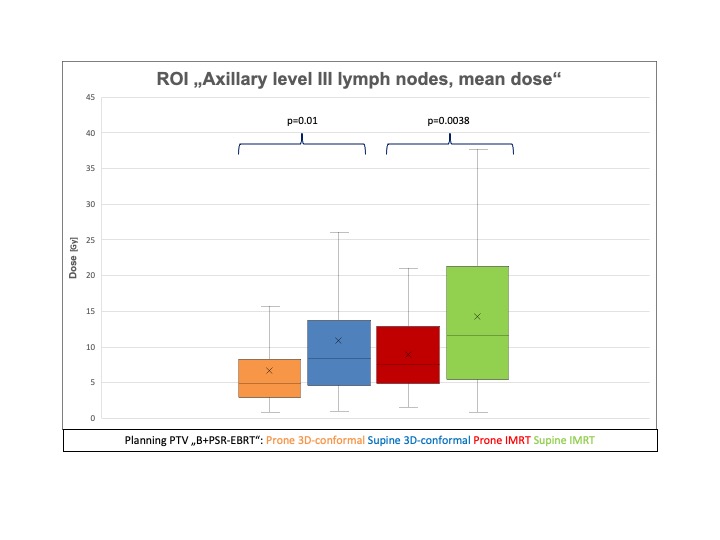


For details on the method of presentation, see the caption for figure S10. The values are given for all patients (n=40). The mean dose in the axillary level III lymphatic region was significantly lower in the prone position compared to the supine position.

In the prone position, 3D EBRT calculated a mean dose of 6.74 Gy (6.67 Gy), IMRT EBRT 9 Gy (SD 5.98 Gy). In the supine position, 3D EBRT resulted in 10.96 Gy (SD 9.05 Gy), and IMRT EBRT in 14.32 Gy (SD 10.76 Gy).

# figure S18: mean dose ROI "Rotter lymph nodes", EBRT of “PTV-B+PSR”


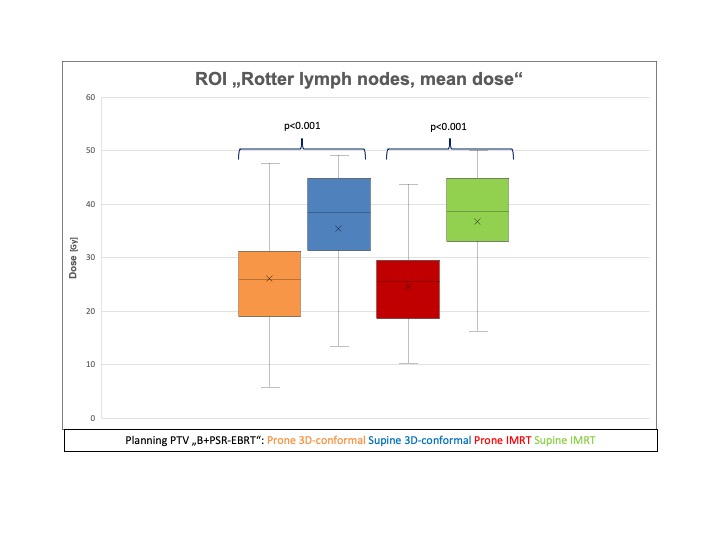


For details on the method of presentation, see the caption for figure S10. The values are given for all patients (n=40). The mean dose in the “Rotter” region was significantly lower in the prone position compared to the supine position.

In the prone position, 3D EBRT calculated a mean dose of 26.2 Gy (SD 10.43 Gy), IMRT EBRT 24.6 Gy (SD 7.82 Gy). In the supine position, 3D EBRT resulted in 35.53 Gy (SD 11.14 Gy), and IMRT EBRT in 36.76 Gy (SD 10.84 Gy).
